# Prevalence and incidence of multiple sclerosis in Bulgaria

**DOI:** 10.3389/fneur.2025.1513390

**Published:** 2025-03-18

**Authors:** Antonia Nikolova, Ivan Milanov, Ksenia Kmetska

**Affiliations:** ^1^Department of Neurology, Medical University, Sofia, Bulgaria; ^2^Multiprofile Hospital for Treatment in Neurology and Psychiatry, Sofia, Bulgaria

**Keywords:** multiple sclerosis, epidemiology, prevalence, incidence, Bulgaria

## Abstract

**Background:**

Multiple sclerosis (MS) is a chronic, inflammatory, autoimmune, demyelinating and neurodegenerative disease of the central nervous system that primarily affects young, active people and is a leading cause of non-traumatic, irreversible neurological deficit. Multiple sclerosis is one of the most studied diseases in neuroepidemiology and is characterized by an uneven geographical distribution worldwide.

**Objective:**

To estimate the prevalence and incidence of multiple sclerosis in Bulgaria and their distribution by age and gender, using data from the latest population census in the country, provided by the National Statistical Institute.

**Methods:**

An epidemiological study, covering a 7-year period—from 2015 to 2021 was conducted in Bulgaria. Eight regions with their population were included in the study—Blagoevgrad, Montana, Pernik, Svoge, Smolyan, Troyan, Haskovo and Shumen. Data, provided by the National Statistical Institute, were used to calculate the values of prevalence and incidence of multiple sclerosis. All cases were diagnosed using the 2017 McDonald’s diagnostic criteria. The results obtained from the study were also used to determine the clinical characteristics of the Bulgarian patient. For the purposes of the epidemiological study an individual questionnaire was developed.

**Results:**

On the prevalence day—07.09.2021, there were 532 people with multiple sclerosis in the studied regions of the country, revealing a prevalence of 121.2/100000 and an incidence of 4.2/100000. 182 of them were males and 350 were females comprising a ratio of 2:1 in favor of the women. More than 50% of all cases had relapsing–remitting course of disease. Secondary-progressive MS had 30% of all patients and 10% suffered from primary progressive multiple sclerosis. Clinically isolated syndrome was present in less than 5% of patients. The mean age at disease onset was 32.2 ± 10.3 years.

**Conclusion:**

The established values of prevalence and incidence position Bulgaria in the area with a high frequency of MS. There is an increase in prevalence and incidence compared to previous studies conducted in the country. The results obtained are similar to those reported by the neighboring countries of the Balkan Peninsula and are close to the average values in Europe according to the latest edition of Atlas of Multiple Sclerosis.

## Introduction

Multiple sclerosis is a chronic, inflammatory, autoimmune, demyelinating and neurodegenerative, socially significant disease of the central nervous system that primarily affects young, active people and is a leading cause of non-traumatic, irreversible neurological deficit (1–3).

Typical of the epidemiology of multiple sclerosis is the presence of a geographic gradient along the North–South axis, which is characterized by the increase in the frequency of the disease with distance from the Equator (4). Kurtzke describes three zones, depending on the frequency of the disease—low, medium and high. The high-frequency zone is characterized by a prevalence rate of 30 and more per 100,000 people and includes the countries of Northwestern Europe, Southern Canada and the North American States. The zone with a medium frequency and a prevalence rate of 5–29 people per 100,000 people includes Southern Europe, the South American States and Australia, and the zone with a low frequency and a prevalence rate of less than 5 per 100,000 people includes the rest of the world (5).

The incidence is higher in high-income countries with a temperate climate, and is prevalent among the white population. Conversely, the disease occurs less often in low-income countries, located in territories with a tropical climate and with a population of predominantly non-Caucasian ethnicities (6).

Multiple sclerosis is one of the most studied diseases in neuroepidemiology. In recent decades, all epidemiological studies have demonstrated an increase in the frequency of multiple sclerosis worldwide by publishing increasingly higher values of the investigated indicators, namely prevalence and incidence (7, 8).

The frequency of multiple sclerosis in North America is among the highest in the world. The overall prevalence for the Americas is estimated at 111/100,000, and the overall incidence is 4.8/100,000 (9). Recent epidemiological studies in Canada show a prevalence of 290/100,000 and an incidence of 14/100,000 (9, 10). The latest data for the United States show a prevalence of 288/100,000 and an incidence of 7.9/100,000 (9).

In the latest edition of the Atlas of Multiple Sclerosis, Europe is presented with a prevalence of 137/100,000 and an incidence of 6.7/100,000 (9). The continent is characterized by high values of these indicators, especially in the Scandinavian countries (11–13). Orkney, Scotland, reported a prevalence rate of 402/100,000, the highest in the world (14). Increasingly high rates of multiple sclerosis incidence and prevalence have also been reported among the countries of the Balkan Peninsula, moving the region out of the low-prevalence zone and into the high-prevalence zone. High values and a sustained upward trend in epidemiological indicators are reported for Bosnia and Herzegovina with a prevalence rate of 91/100,000 (9, 15, 16), Serbia with prevalence of 136/100,000 an incidence of 3.4/100,000 (9, 17–19), and Croatia—143.8/100,000 (20). Data for Romania date from the 1990s and report incidence rates ranging from 22.1/100,000 in Transylvania to 46.4/100,000 in Bucharest, with an overall national incidence of around 35/100,000 (21).

There is an increasing trend in prevalence and incidence of the disease in Southern Canada; the northern parts of the United States; Northern, Southern and Southeastern Europe. The incidence of the disease is increasing among females and African Americans. Based on these data, a number of geographic areas that previously fell into the low-and medium-frequency zones of the disease now fall into the high-frequency zone. Moreover, prevalence and incidence in some high-frequency areas continues to increase (7, 22).

A geographic gradient in the incidence of multiple sclerosis has been observed, with incidence decreasing from north to south in North America and Western Europe and increasing from north to south in Australia. Although there is a tendency for this gradient to decrease, it is still present, and Kurtzke’s division of geographic areas into low-, medium-, and high-incidence areas is still valid, albeit with different values (22).

In recent decades, a number of authors have disputed the presence of such a geographic gradient in the Northern Hemisphere and its preservation in the Southern Hemisphere (23, 24). The exact reasons for the differences in the geographic distribution of multiple sclerosis are not clear. It depends on the interaction between the genetic pool of individuals and environmental factors. This uneven distribution of the disease predetermines a leading role of environmental factors, which is also supported by migration studies (4, 25).

The latest edition of the Multiple Sclerosis Atlas shows an increase in the number of people with multiple sclerosis worldwide to 2.8 million in 2020 and 2.9 million people in 2023. Multiple sclerosis affects not only adults but also children, with at least 30,000 people under the age of 18 having been diagnosed with multiple sclerosis worldwide. The global prevalence of multiple sclerosis is estimated to be 37 per 100,000 (9).

The first studies of the prevalence of multiple sclerosis in Bulgaria date back to the 1960s and cover the period 1952–1956, revealing a prevalence of 5.9/100000 (26–28). Two detailed epidemiological studies of the prevalence of multiple sclerosis have been conducted in the country. The first covered the period 1970–1979. The prevalence day in this study was 31/12/1979 and the estimated prevalence was 21.3/100000 (29). The second study was conducted during the period between 1995 and 1998 and revealed a prevalence of 44.5/100000 (30).

There are no more recent data available.

The aim of our study is to provide new and contemporary information on the epidemiology of multiple sclerosis in Bulgaria.

## Methods

An epidemiological study covering a 7-year period—from 2015 to 2021—was conducted in Bulgaria. Eight regions with their population were included in the study—Blagoevgrad, Montana, Pernik, Svoge, Smolyan, Troyan, Haskovo and Shumen. All of them (except Svoge and Troyan) are administrative centers of the regions of interest. The epidemiological indicators of prevalence and incidence in individual regions and their distribution by age and gender were determined in order to study the frequency of multiple sclerosis in the country. Data from the latest population census in the country—07.09.2021, provided by the National Statistical Institute, were used to calculate the values of prevalence and incidence of multiple sclerosis.

The results obtained from the study were also used to determine the clinical characteristics of the Bulgarian patient.

**Figure 1:**
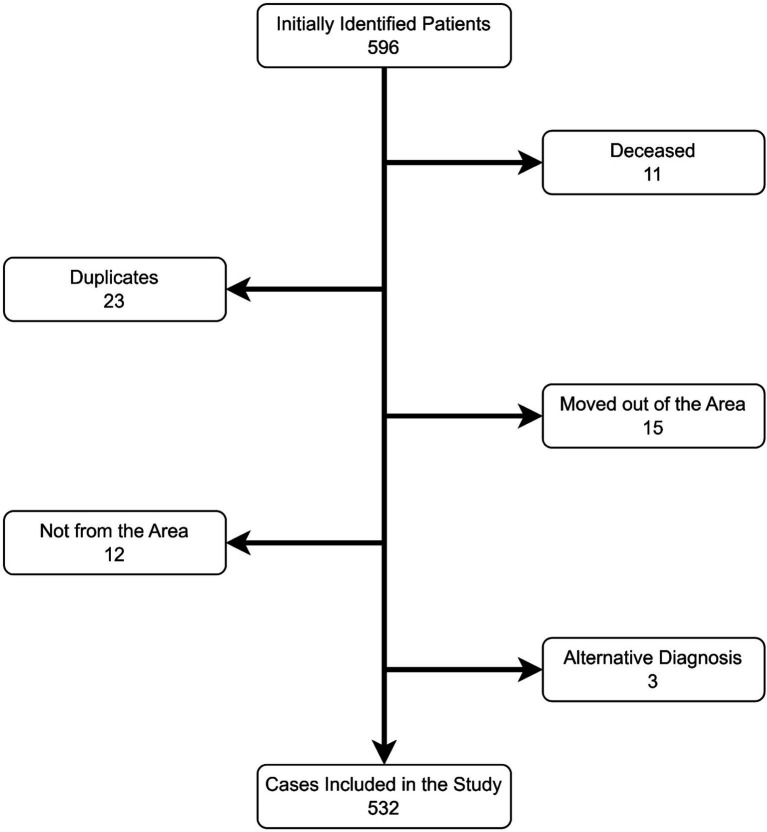
Cases ascertainment flowchart.

To determine the cases, we used documentation provided by the hospital system of the St. Naum Hospital, the National Health Insurance Fund, the Regional Health Insurance Fund by municipalities, the Regional Health Inspections with information on the issued decisions of the Territorial Expert Medical Commissions, hospital registers, private and state medical centers, outpatient practicing neurologists and patient organizations. All collected information was summarized and analyzed, duplicates and deceased patients were eliminated, and a list of patients in all municipalities was compiled. During the gathering of information about the cases of multiple sclerosis, we eliminated 11 deceased patients, 23 duplicates (one and the same information was available in more than one database), 16 had moved out of the area, 12 of them lived in another area (Figure 1).

For the purposes of our epidemiological study an individual questionnaire was developed. A detailed medical history record was taken and a thorough somatic and neurological examination was performed. Information provided in the form of imaging studies (Magnetic Resonance Tomography), neurophysiological studies (evoked potentials, optical coherence tomography) and cerebro-spinal liquid after lumbar puncture were analyzed. Lumbar puncture was performed on 383 (72%) of the cases, with positive oligoclonal bands found in 348 of them (91%). The remaining participants refused the procedure, including those diagnosed with clinically isolated syndrome. Our aim was to gather and analyze as much information as possible in order to avoid misdiagnosis. A broad and thorough differential diagnosis was made to ensure that only patients with multiple sclerosis were included in the study. Two patients were excluded because they were diagnosed with NMOSD (Neuromyelitis optica spectrum disease) and one more patient was excluded due to the genetic verification of CADASIL (cerebral autosomal dominant arteriopathy with subcortical infarcts and leukoencephalopathy).

All cases were diagnosed using the 2017 McDonald’s diagnostic criteria.

The phenotypic course of multiple sclerosis was determined according to the latest classification of 2013. A specialized statistical package SPSS (Statistical Package for the Social Sciences) version 20.0 was used to process the survey data.

## Results

On the prevalence day—07.09.2021, there were 532 people with multiple sclerosis living in the studied municipalities. 182 of them were men and 350 were women, comprising a ratio of 2:1 in favor of the women. All cases of the disease were diagnosed using the 2017 McDonald’s diagnostic criteria and the diagnosis was confirmed in a university hospital for all cases. Only residents of the studied regions were included in the study.

The prevalence in different regions varied from 116.8/100000 in Haskovo to 124.5/100000 in Pernik, with a national average of 121.2/100000. In all regions the prevalence was higher among the female sex. The highest prevalence among women was found in Troyan and had a value of 172.8/100000, while the highest prevalence among men was found in Smolyan—105.7/100000 (Table 1). Most women were in the age group of 40–49 on the territory of Blagoevgrad, Montana, Pernik, Smolyan, Troyan and Shumen. In Svoge, the number of women suffering from the disease was greatest in the age group of 50–59 years, and in Haskovo—30-39 years. A slightly different distribution was discovered for the male gender. In Blagoevgrad, Montana, Troyan and Haskovo, the greatest percentage of males with the disease fell within the age group 40–49. In Shumen, the disease was most common in men aged 30–39. In Pernik and Smolyan, the disease was found most commonly among males in the age range of 20–29 years, while in Svoge the most men with the disease were between 50 and 59 years of age.

**Table 1:** Prevalence per 100,000 in the studied regions.

Region	Cases (N)			Population			Prevalence (per 100,000)		
	Male	Female	Total	Male	Female	Total	Male	Female	Total
Blagoevgrad	32	51	83	33,181	35,897	69,078	96.4	142.1	120.2
Montana	22	33	55	22,476	23,795	46,271	97.9	138.7	118.9
Pernik	34	71	105	40,232	44,115	84,347	84.5	161	124.5
Svoge	7	17	24	9,578	9,849	19,427	73.1	172.6	123.5
Smolyan	17	24	41	16,090	17,598	33,688	105.7	136.4	121.7
Troyan	9	24	33	12,993	13,891	26,884	69.3	172.8	122.7
Haskovo	30	65	95	38,470	42,872	81,342	78	151.6	116.8
Shumen	34	62	96	37,919	41,248	79,167	89.7	150.3	121.3

Furthermore, we determined the number of new cases of the disease which appeared in the studied municipalities during the studied period. The appearance of the first symptoms, rather than the year of diagnosis, was interpreted as the onset of the disease, for some patients exhibited a long interval between the two events. The annual incidence was highest in Smolyan—5.5/100000. Here was also the highest incidence among men—4.4/100000, while the incidence among women was highest in Troyan—7.2/100000 (Table 2). In all studied municipalities, the incidence was highest in the age range 20–29 years, with the exception of Haskovo, where the age group 30–39 years exhibited the highest incidence. In the sex-specific incidence, the highest value for women was 55.9/100000 and was found in Svoge, while the incidence for men was highest in Montana in the age group 20–29 years and had a value of 22.4/100000. The average values of the two epidemiological indicators for the country were as follows: a prevalence of 121.2/100000 and an incidence of 4.2/100000.

**Table 2:** Annual incidence per 100,000 in the studied regions.

Region	New Cases (N)			Population			Annual incidence (per 100,000)		
	Male	Female	Total	Male	Female	Total	Male	Female	Total
Blagoevgrad	5	12	17	33,181	35,897	69,078	2.2	4.8	3.5
Montana	5	9	14	22,476	23,795	46,271	3.2	5.4	4.3
Pernik	8	16	24	40,232	44,115	84,347	2.8	5.1	4.1
Svoge	1	4	5	9,578	9,849	19,427	1.5	5.8	3.7
Smolyan	5	8	13	16,090	17,598	33,688	4.4	6.5	5.5
Troyan	2	7	9	12,993	13,891	26,884	2.2	7.2	4.8
Haskovo	7	15	22	38,470	42,872	81,342	2.6	5	3.9
Shumen	9	11	20	37,919	41,248	79,167	3.4	3.8	3.6

The mean age of all study participants was 43.78 ± 12.12 years with a minimum value of 13 years and a maximum of 75 years. In males, the average age was 42.80 ± 11.87, whereas in females—44.30 ± 12.24 years.

The mean age at disease onset was 32.2 ± 10.3 years, varying between 11 and 52 years. The average age at the onset of the relapsing–remitting form of the disease was 29.4 ± 8.69, and of the primary-progressive form—47 ± 9.24 years.

In 55.6% of cases, the diagnosis of the disease was made in the first 6 months after the appearance of the first symptoms, and in 34%—in the first month. The progressive forms of the disease were diagnosed between 1 and 2 years after onset.

Patients were grouped according to the clinical course of multiple sclerosis—relapsing–remitting (PRMS), secondary-progressive (SPMS), primary-progressive (PPMS) and clinically isolated syndrome (CIS). In the highest percentage of cases, more than 50%, the disease had relapsing–remitting course.

In each of the studied regions, more than 50% of the cases had relapsing–remitting course. The secondary-progressive course of disease follows (≈30%). The lowest percentage was observed in patients with a primary progressive course (≈10%) and patients with a clinically isolated syndrome (≈5%).

The highest percentage of relapsing–remitting multiple sclerosis was observed in Blagoevgrad, Montana and Troyan—about 60%. The secondary-progressive course of disease was the second most common and its highest value was found in Svoge—38%. PPMS and CIS were found to have the lowest percentages. Most patients with a primary-progressive course of the disease were found in Haskovo—about 12% of all cases. Montana was the municipality home to the most patients with a clinically isolated syndrome—6% of all cases in the municipality (Table 3).

**Table 3:** Clinical course of disease in the studied regions.

		Course of disease				Total
		CIS	RRMS	SPMS	PPMS	
Blagoevgrad	N	2	50	22	9	83
	%	2	60	27	11	100
Montana	N	3	33	15	4	55
	%	6	60	27	7	100
Pernik	N	5	58	31	11	105
	%	5	55	30	10	100
Svoge	N	1	12	9	2	24
	%	4	50	38	8	100
Smolyan	N	2	21	14	4	41
	%	5	51	34	10	100
Troyan	N	1	20	10	2	33
	%	3	61	30	6	100
Haskovo	N	5	51	27	12	95
	%	5	54	29	12	100
Shumen	N	5	50	33	8	96
	%	5	52	35	8	100
Mean EDSS		2.5	3.5	5.5	6.0	

The number of cases of the disease starting in childhood was greatest in the municipalities of Shumen—16%, as well as Montana and Svoge—13%. Most commonly, the onset of the disease occurred in between 20 and 29 years of age. The largest percentage of patients with onset in the 20–29 age group was found in Smolyan—44%, and the largest percentage of patients with the debut of multiple sclerosis in the age of 30–39 was observed in Montana—38% (Table 4).

**Table 4:** Age of onset of disease in the studied regions.

Region		Age of onset					Total
		0–18	19–29	30–39	40–49	50–59	
Blagoevgrad	N	8	28	30	15	2	83
	%	10	34	36	18	2	100
Montana	N	7	20	21	5	2	55
	%	13	36	38	9	4	100
Pernik	N	8	41	31	16	9	105
	%	8	39	30	15	8	100
Svoge	N	3	9	6	5	1	24
	%	13	38	25	21	4	100
Smolyan	N	2	18	12	7	2	41
	%	5	44	29	17	5	100
Troyan	N	3	11	9	6	4	33
	%	9	34	27	18	12	100
Haskovo	N	8	31	26	19	11	95
	%	8	33	27	20	12	100
Shumen	N	15	29	31	17	4	96
	%	16	30	32	18	4	100

The most common first symptoms of the disease were visual and sensory disturbances, respectively—24.7 and 24.3% of all cases. This was followed by brain stem disorders, exhibited by 13.7% of cases, a polysymptomatic onset (12.6%), coordination disorders (12.1%) and movement disorders—10.9%. The rarest first symptoms were cognitive and mental disorders—0.2%. Regarding the finding of the neurological status, motor disorders were the most common (80.6%), followed by sensory (76.9%), coordination (75.9%) and pelvic-reservoir disorders (66.4%).

## Discussion

On the prevalence day—07.09.2021, there were 532 people with multiple sclerosis in the studied regions of the country, revealing a prevalence of 121.2/100000 and an incidence of 4.2/100000. 182 of them were males and 350 were females comprising a ratio of 2:1 in favor of the women. More than 50% of all cases had relapsing–remitting course of disease. Secondary-progressive MS had 30% of all patients and 10% suffered from primary progressive multiple sclerosis. The mean age at disease onset was 32.2 ± 10.3 years.

Bulgaria is a small country located north of the equator, in the eastern part of the Balkan Peninsula, in Southeastern Europe. Its territory extends from 41°14’ N to 44°12’ N and from 22°21′ E to 28°36′ E.

The first study of the epidemiology of multiple sclerosis in Bulgaria covered the period 1952–1959. It reported a prevalence of 5.9/100000 (26). Another study focused on the capital city Sofia yielded a prevalence of multiple sclerosis of 30.2/100000 in 1992, significantly higher than the total prevalence for the country (31). In the period 1993–1995, Milanov et al. investigated the epidemiology of multiple sclerosis in two small districts, Svoge and Troyan, and estimated a prevalence of 39.3/100000 and 39.1/100000, respectively (28). To date, two detailed studies of the epidemiology of multiple sclerosis in the country have been conducted. The first one covered the period of 1970–1979. The prevalence day in this study was 31/12/1979 and the estimated prevalence was 21.3/100000 (29). The second, and last published study encompassed the years between 1995 and 1998. It estimated a prevalence of 44.5/100000 and an incidence 1.03/100000 (30). Bulgarians exhibited a higher prevalence of multiple sclerosis than Gypsies (32).

Bulgaria was positioned in the areas with low and medium frequencies of the disease and a permanent increasing tendency in the frequency of multiple sclerosis in Bulgaria has been established. Such a trend is also present in the other countries of the Balkan peninsula (17, 19, 21, 33), Southeastern and Central Europe (13, 20, 34, 35).

For a period of nearly 25 years, there has been a lack of up-to-date epidemiological data on the prevalence of multiple sclerosis in Bulgaria. We conducted a complex clinical and epidemiological study of multiple sclerosis in the country which included the municipalities Blagoevgrad, Montana, Pernik, Svoge, Smolyan, Troyan, Haskovo and Shumen. Svoge and Troyan were included in the study due to the availability of prior data regarding the epidemiology of multiple sclerosis in them. They make it possible to compare and analyze the results. The other regions were selected from all over the country in order to ensure representative results and allow for analysis of the epidemiology of the disease throughout the country as accurate as possible. Three of the studied regions (Montana, Troyan and Shumen) are located in Northern Bulgaria, another three (Blagoevgrad, Smolyan and Haskovo) in Southern Bulgaria, and two of the cities (Pernik and Svoge) are located near the capital Sofia, with Pernik being among the largest cities in the country, and Svoge being a smaller, more rural region. The identified cases were investigated and analyzed with respect to a number of indicators such as a phenotypic form of multiple sclerosis, first symptoms and mean age at disease onset, time to diagnosis, neurological status findings and EDSS score calculation.

In 2013 the international scientific community proposed and accepted a clear description and definition of the different clinical forms of MS—relapsing–remitting, primary progressive, and secondary progressive multiple sclerosis (36). A new clinical category, clinically isolated syndrome (CIS), is introduced. This term refers to the first episode of inflammatory demyelination, suspected of multiple sclerosis, in which dissemination of the process over time cannot be demonstrated (37). The relapsing–remitting form is the most common and is observed in about 85% of patients (38). After a varying period of time, untreated patients with the relapsing–remitting form of MS progress to a secondarily progressive form of the disease (39). The primary progressive form is characterized by a slow and gradual accumulation of irreversible neurological deficits from the onset of the disease. It occurs in about 10% of patients (40).

In our study as well the most common phenotypic form of the disease was relapsing–remitting multiple sclerosis—in 55.5% of cases, followed by secondary-progressive multiple sclerosis (30.1%) and primary-progressive multiple sclerosis with 9.6%. Clinically isolated syndrome was present in 4.9% of cases. The mean age at disease onset was 32.2 ± 10.3 years. The average age at the onset of the relapsing–remitting form of the disease was 29.4 ± 8.69, and of the primary-progressive form—47 ± 9.24 years. The beginning of relapsing–remitting form of the disease was most often with sensory (29.6%) and visual (25.2%) disorders, while motor disorders were most often the first symptom in the primary-progressive form. In a larger percentage of patients, the neurological deficit, assessed using the EDSS scale, is between 2 and 4–45.1% of all cases. 17.9% of patients have score > 6.

In the early 20th century, the prevailing belief was that multiple sclerosis was a disease that predominated among males (6). Then, in the 1940s, a sustained trend toward a change in the male–female ratio began to be observed, and in recent years, number of studies from different countries (Canada, Germany, France, Norway, Denmark) documented a clear increase in the prevalence and incidence of multiple sclerosis among females (23, 41).

In our study as well the disease occurred more often among the females, with the ratio between the two sexes being 2:1 in favor of the women.

Everything mentioned above proves that in Bulgaria there is no different pattern in terms of the course of the disease and its spread among the sexes compared to other countries and populations.

Traditionally, near the capital Sofia, as well as in the capital itself, high rates of prevalence have been established in a number of previous studies in the country. The authors explain this fact with the development of industrialization, better health culture of the population and improvement of health care and services with higher qualification of medical specialists in the capital (31). The study of prevalence in the capital, however, is hampered by the presence of numerous medical facilities working with patients suffering from multiple sclerosis and the significant migration flow, which create a prerequisite for missing or duplicated cases and was the main reason why Sofia was not included in our study. Comparing the individual municipalities, we found the highest value of age-specific prevalence—409.3/100000—in Svoge for the age group of 50–59 years. The highest value of age-specific prevalence in females was also in the age group 50–59 and again in Svoge—519.5/100000. In the previous epidemiological study in the country from 1992 to 1997, the prevalence among women in Svoge was also highest in the age group 50–59, but the reported values were significantly lower—171.5/100000 (30). No latitudinal gradient was found, probably due to the small territory of the country. The high prevalence rate in the 50–59 age group in Svoge can be explained with the aging population in this region because young peaple migrate to the near bigger cities.

We also found high values of the epidemiological indicator of incidence. In the different regions, the values varied between 3.5 and 5.5, with the average incidence for the country being 4.2/100000. Among females, the highest incidence was found in Troyan—7.2/100000 and for males in Smolyan—4.4/100000. The established differences in the values of prevalence and incidence could be explained by the differences in the gender and age structure of the population in the studied areas.

For comparison, in the study from 1970 to 1979, the incidence in the country was below 1/100,000 (29), and the study from 1992 to 1997 reported an incidence of 1.03/100000 (30). The overall prevalence of 121.2/100000 and overall incidence of 4.2/100000 in our study reveal a well-defined trend of progressive increase in the frequency of multiple sclerosis in Bulgaria during the last decades. The prevalence is about 3 times higher and the incidence 4 times higher than the reported values from the previous epidemiological survey covering the period 1992–1997 and published in 1999 (30). The results obtained are similar to those reported by the neighboring countries of the Balkan Peninsula (17, 19, 33) and are close to the average values in Europe according to the latest edition of the Atlas of Multiple Sclerosis, where an average prevalence of 137/100000 and incidence of 6.7/100000 is reported (9).

The increasing values of prevalence and incidence of multiple sclerosis in Bulgaria can be explained by an increased life expectancy as a result of improved health care services, the early and precise diagnosis of patients using modern diagnostic criteria and the active search for patients due to the possibilities of modern disease-modifying therapy, as well as a real increase in the number of new cases of the disease (42).

Our study was designed to investigate current trends in the epidemiology of multiple sclerosis in Bulgaria because for a period of more than 25 years, there has been a lack of up-to-date epidemiological data in the country. A clear increase in the prevalence and incidence of MShas been established similar to the other countries of the Balkan Peninsula, Southeastern and Central Europe. Limitation of the study is the lack of information for the epidemiology of multiple sclerosis over the whole country. The results warrant further research, encompassing all regions of the country, for the appropriate allocation of human, social and financial resources.

## Data Availability

The original contributions presented in the study are included in the article/supplementary material, further inquiries can be directed to the corresponding author.
